# MiR-638 acts as a tumor suppressor gene in gastric cancer

**DOI:** 10.18632/oncotarget.22567

**Published:** 2017-11-20

**Authors:** Yu Shen, Haiqun Chen, Ling Gao, Weigang Zhang, Jun He, Xiaohua Yang, Lei Qin, Xiaofeng Xue, Zhaoji Guo

**Affiliations:** ^1^ Department of General Surgery, The First Affiliated Hospital of Soochow University, Suzhou 215006, P.R. China; ^2^ Department of General Surgery, Xinhua Hospital Affiliated to Jiaotong University Chongming Branch, Shanghai 200000, P.R. China

**Keywords:** miR-638, SOX2, gastric cancer, cell proliferation, invasion

## Abstract

Gastric cancer is one of the major causes of cancer mortality. Several microRNAs play a role in the tumor growth and invasion. However, the underlying molecular mechanism remains poorly understood. We detected the miR-638 expression levels in tumor samples and adjacent noncancerous tissues from 68 patients with gastric cancer as well as in the gastric cancer cell line SGC-7901 and SC-M1. The cell cycle was analyzed by flow cytometry, cell proliferation was observed by CCK-8 assay and cell invasion was detected using Transwell assay. MiR-638 was down-regulated in human GC tissues and its expression level was negatively correlated to TNM stage and lymph metastasis. In the cell lines, aberrant expression of miR-638 was related to the cell proliferation, cell cycle and invasion. We also found that SOX2 had a negative correlation with miR-638 in GC tissues, and miR-638 overexpression could decrease SOX2 expression level by directly binding the 3’-UTR of SOX2. *in vitro*, down-regulating SOX2 by siRNA could counteract the effect of miR-638 inhibitor on GC cells proliferation and invasion. Our results demonstrate that miR-638 may play a pivotal role in the growth and invasion of GC.

## INTRODUCTION

Gastric cancer (GC) makes a significant contribution to the global health burden, and it is the fifth most frequently occurring cancer. Moreover, GC is the third most common cause of cancer-related mortality More than 950,000 new patients are diagnosed with GC every year, and approximately 720,000 patients died from GC in 2012 [1]. Currently, surgical resection is the most recommended and effective treatment for GC. Besides, early radical resection is vital for decreasing the mortality associated with this disease. GC diagnosed at advanced stages is associated with a poor prognosis [2].

Although some progress has been made to find the key factors in the occurrence and progression of GC, we still need to explore further mechanisms how the key factors act to promote tumor progression. MicroRNAs (miRNAs) are short RNA molecules (18-25 nucleotides in length) that belong to the family of small non-coding RNAs [3]. MiRNAs have been found to participate in the regulation of gene expression and play important roles in a vast range of fundamental process such as early development, cell differentiation, proliferation, apoptosis, and stress response [4, 5], relying on the complementarities between the limited region of sequence at the 5′-end of the miRNA (the “seed”) and the 3′-untranslated region (UTR) of specific target mRNAs [6]. Thus far, 2588 mature miRNAs have been identified in humans, and many of these miRNAs have been found to contribute to the initiation, progression, and prognosis of many cancers [7]. It is remarkable that some miRNAs can function inconsistently depending on the cell types of cancers and pattern of gene expression [8–10].

Previous microarray studies have demonstrated eight miRNAs, namely, miR-21, miR-25, miR-92, miR-223, miR-106b, miR-17, miR-18a, and miR-20a, which were consistently reported to be up-regulated in GC tissues. In addition, miR-375, miR-378, and miR-638 were revealed to be the most commonly down-regulated miRNAs [11, 12], but more *in vivo* and *in vitro* evidence is required to confirm the role of these miRNAs in GC.

In this study, we intended to explore the role of miR-638 in GC. Although miR-638 has been reported to be of inhibitory effect in the GC cell proliferation by other groups [13, 14], we found it also could regulate the invasion of GC by targeting SOX2, a factor that can regulate the self-renewal of stem cells and induce stem cell-like features of cancer cells.

## RESULTS

### miR-638 is down-regulated in human GC tissues

**Figure 1:**
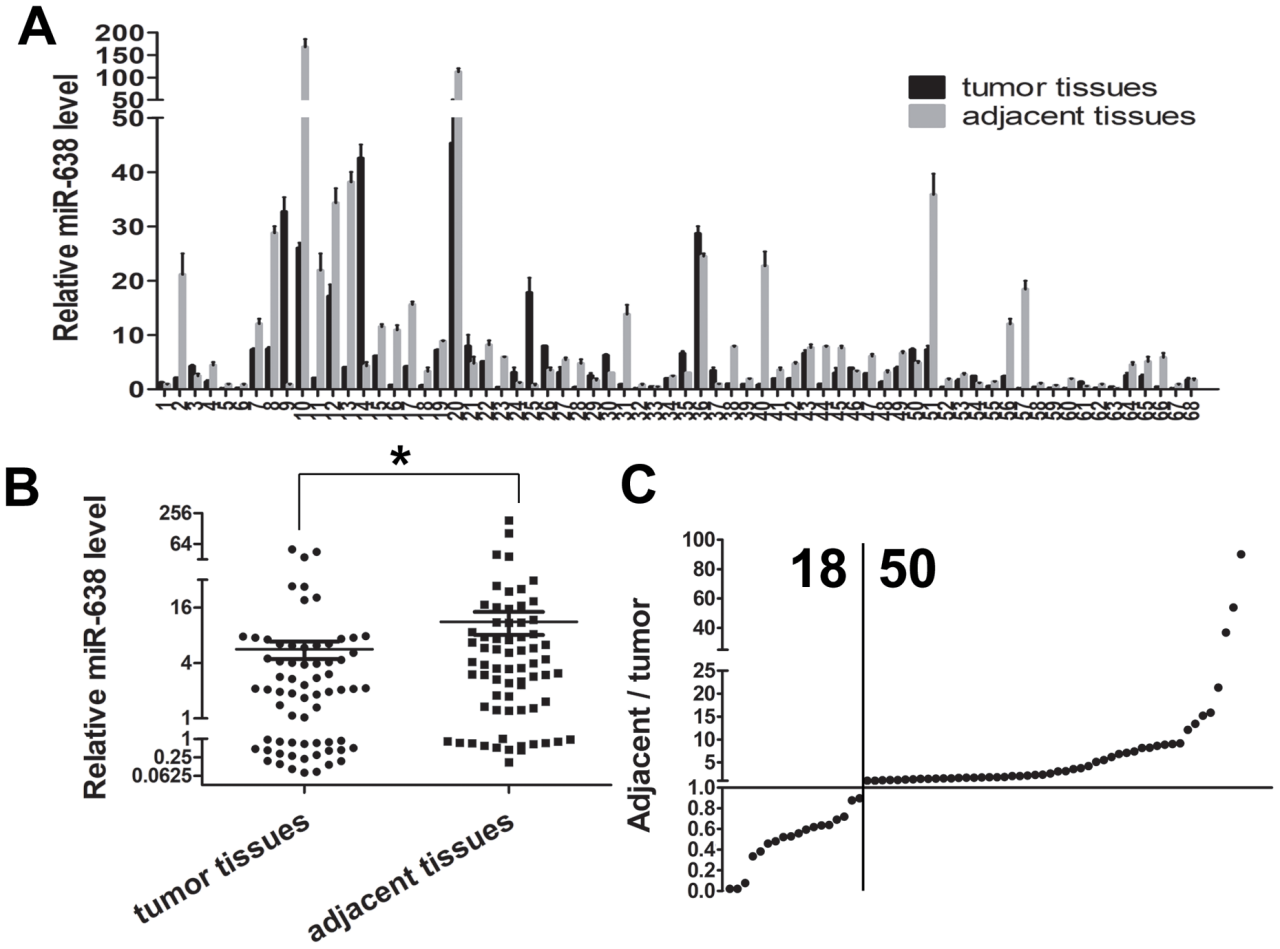
MiR-638 expression was down-regulated in human GC tissues **(A)** miR-638 expression levels in 68 pairs of GC and matched adjacent gastric tissues were analyzed by qRT-PCR. **(B)** A scatter plot showed the result of miR-638 expression in the cancer tissues and adjacent tissues. **(C)** miR-638 expression was down-regulated in 50/68 of the cancer samples. ^*^ indicates significant difference compared with control group (p < 0.05).

To evaluate the expression level of mature miR-638, we conducted qRT-PCR in GC tissues and matched adjacent tissues from 68 cases of GC patients. Significant lower miR-638 level was detected in 50 of the 68 GC tissues compared with adjacent tissues (Figure 1A and 1C), and relative average expression level of miR-638 was 5.637±1.214 in tumor specimens vs 11.220±3.148 in adjacent tissues. (p <0.05, Figure 1B)

A correlation analysis between the miR-638 expression level and the clinicopathological characteristics of patients with GC was presented in Table 1. MiR-638 expression was lower in patients with high TNM stage (III–IV) than those with low stage (I–II) (P<0.05). Moreover, miR-638 expression was negatively correlated to lymph metastasis in GC (P<0.05). However, miR-638 expression level was not correlated with age, sexuality, tumor stage, degree of differentiation, or distant metastasis in the GC samples (Table 1). All these results of correlation analysis indicated that miR-638 might take part in the metastasis of GC.

**Table 1:** A correlation analysis between the miR-638 expression level and the clinicopathological characteristics

Clinical characteristics	Patients	Low miR-638 expression in tumor(<Median)	High miR-638 expression in tumor(>Median)	*P* value
Age (years)	68			
<60	21	9	12	
>=60	47	25	22	0.4310
Gender				
Male	56	25	31	
Female	12	9	3	0.0563
T stage				
T1-T3	26	10	16	
T4	42	32	30	0.6813
Tumor differentiation				
High-moderate	42	19	23	
Poor-undifferentiation	6326	15	11	0.3182
TNM stage				
I, II	30	10	20	
III, IV	38	24	14	0.0146^*^
Distant metastasis				
No	60	31	29	
Yes	8	3	5	0.4516
LN metastasis				
No	28	10	18	
Yes	40	24	16	0.0487^*^

### miR-638 regulates cell proliferation and cell cycle *in vitro*

**Figure 2:**
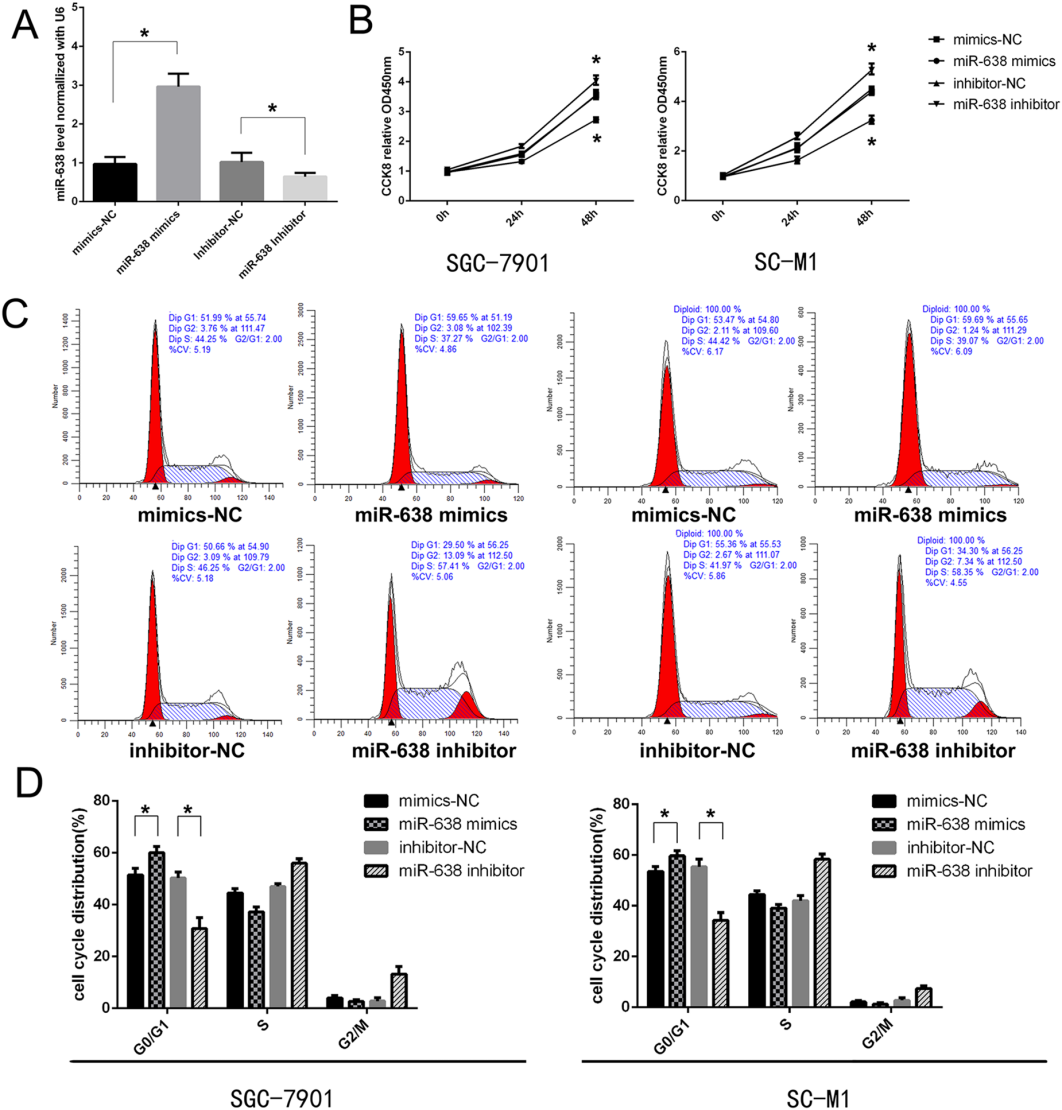
MiR-638 suppressed cell growth and induces cell cycle arrest in GC cells **(A)** miR-638 expression in cell lines transfected with miR-638 mimics, miR-638 inhibitor and controls was determined by a qRT-PCR assay. **(B)** Cell proliferation was measured using the CCK-8 assay at the indicated time points, and a growth curve was plotted. **(C** and **D)** Flow cytometry assay was performed to assess cell cycle change of GC cell lines after transfected with miR-638 mimics, miR-638 inhibitor and controls. ^*^ indicates significant difference compared with control group (p < 0.05).

To evaluate the role of miR-638 in the development of GC, gain- or loss-of-function tests were performed by transfecting SGC-7901 and SC-M1 with miR-638 mimics, inhibitor and corresponding controls. The transfection efficiency was confirmed through qRT-PCR after 24 h (Figure 2A). To detect whether miR-638 could regulate the proliferation of GC cell lines *in vitro*, the effect of miR-638 on cell proliferation was investigated by CCK-8 analysis. We found that the proliferation ability of GC cells was impaired after transfected with the miR-638 mimics 48h. Meanwhile, miR-638 inhibition could promote cell proliferation (p < 0.05, Figure 2B). We also applied flow cytometry to study the effect of miR-638 on cell cycle regulation. The results revealed that miR-638 mimics significantly induced cell cycle arrest in the G0/G1 phase compared to the control group. Furthermore, the number of G0/G1 phase cells was remarkably decreased in the miR-638-inhibitor group relative to the control group (p < 0.05, Figure 2C and 2D).

### miR-638 changed the invasiveness of GC cell lines *in vitro*

**Figure 3:**
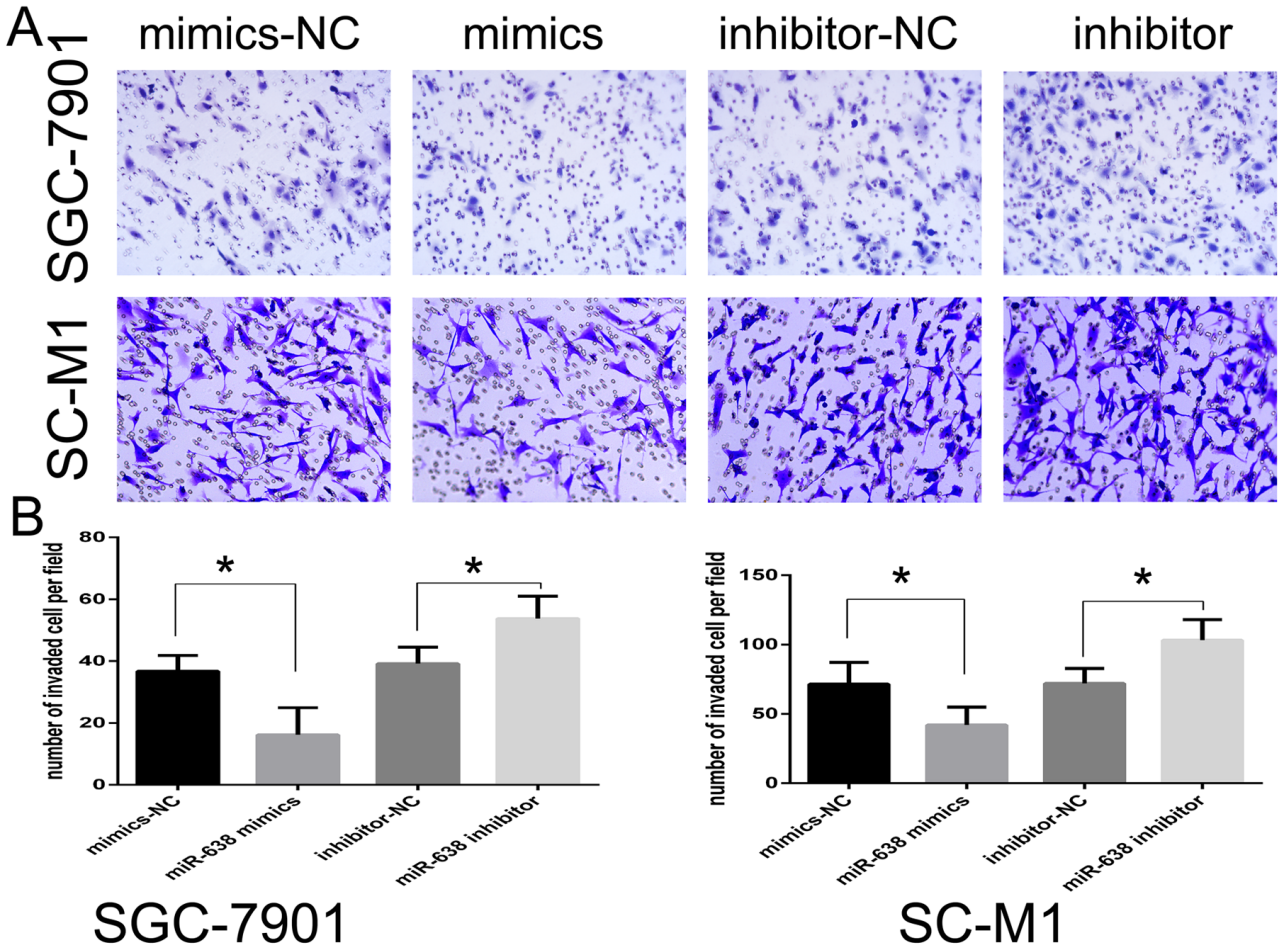
MiR-638 inhibited the invasive ability of GC cell lines **(A)** Transwell assay was performed after GC cell lines were transfected with miR-638 mimics, miR-638 inhibitor, NC and inhibitor NC for 24 h. The representative images of invasive cells at the bottom of the membrane stained with Giemsa were presented as shown. **(B)** The quantification of invasive cells was presented as number of invaded cells per field, magnification, ×20. (^*^ represents p < 0.05).

We further estimated the effect of miR-638 on cell invasion using the Transwell invasion assay after transfection with miR-638 mimics or inhibitor. MiR-638 up-regulation decreased the invasive ability of SGC-7901 and SC-M1 cells (p < 0.05), while miR-638 down-regulation increased the invasive ability of SGC-7901 and SC-M1 cells (p < 0.05; Figure 3A and 3B).

### SOX2 expression was lower in GC and regulated directly by miR-638

**Figure 4:**
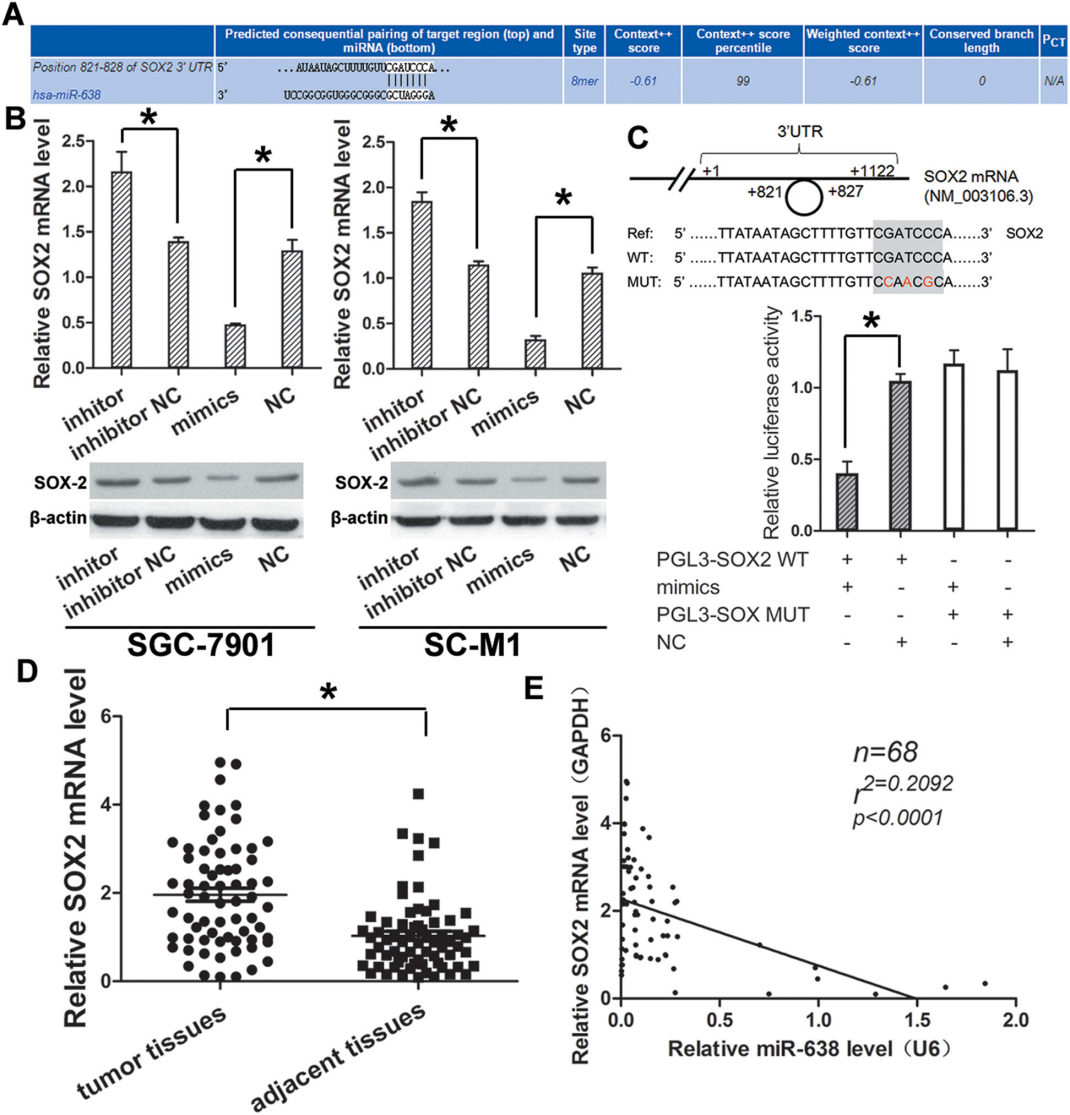
SOX2 expression was higher in GC and could be regulated directly by miR-638 **(A)** Initial screening result of miR-638 target gene by bioinformatics prediction. **(B)** The predicted gene SOX2 was validated by qRT-PCR and western blot in GC cell lines transfected with miR-638 mimics, miR-638 inhibitor, NC and inhibitor NC. **(C)** Schematic of the wild-type and mutant 3’-UTRs of the SOX2 vector constructs was presented. The relative luciferase activity assays were performed after wild-type (WT) or mutant-type (MT) pMir luciferase vector co-transfected with miR-638 mimics or NC. **(D)** The expression level of SOX2 in the 68 pairs of GC and matched adjacent gastric tissues were detected by qRT-PCR. **(E)** A negative correlation was found between expression level of miR-638 and SOX2 in GC samples. (^*^ indicates p < 0.05).

We used the TargetScan databases (Figure 4A) to search for potential targets for miR-638, and found some potential genes from these databases. Of these targets, SOX2 were significantly down-regulated after transfecting GC cell lines SC-M1 and SGC-7901 with miR-638 mimics, confirmed by qRT-PCR (Figure 4B). Therefore, SOX2 may be the candidate tumor-related targets of miR-638.

What's more, we performed Western blot assay to confirm that whether the change of miR-638 expression could affect the expression of SOX2 *in vitro*. It showed that SOX2 was negatively regulated by miR-638 both in SC-M1 and SGC-7901 cell lines. MiR-638 mimics could decrease SOX2 expression; meanwhile, miR-638 inhibitor could increase SOX2 expression (Figure 4B).

To further examine the relationship between miR-638 and SOX2, the wild 3′-UTR fragment containing the predicted site of SOX2 (pMir–SOX2) and its corresponding mutant sequence (pMir–SOX2–MUT) as a control group were cloned into pMir luciferase vector. Luciferase activity assay showed that the luciferase intensity in pMir–SOX2 was suppressed by miR-638 mimics, but not in pMir–SOX2–MUT vector (Figure 4C). The result indicated that SOX2 could be down-regulated by miR-638 through directly binding to its 3′ -UTR.

Furthermore, the expression level of SOX2 were detected in the GC tissues and matched adjacent normal tissues (n = 68) as above by using qRT-PCR. Significantly lower SOX2 (P < 0.05, Figure 4D) RNA level were detected in cancer tissues compared with adjacent tissues. Moreover, by correlation analysis, a significant negative correlation was observed between miR-638 and SOX2 (R = 0.2092; P < 0.001, Figure 4E) in cancer tissues.

### Knocking-down SOX2 with siRNA reversed the promotion of GC cells proliferation and invasion caused by down-regulation of miR-638

In the above, we have confirmed the inhibitory effect of miR-638 on proliferation and invasion of GC cell lines. Here, to further explore whether the effect of miR-638 was dependent on SOX2, we co-transfected miR-638 inhibitor and siRNA of SOX2 in GC cell lines (SC-M1 and SGC-7901) depending on the mentioned result that inhibition of miR-638 could up-regulate SOX2.

**Figure 5:**
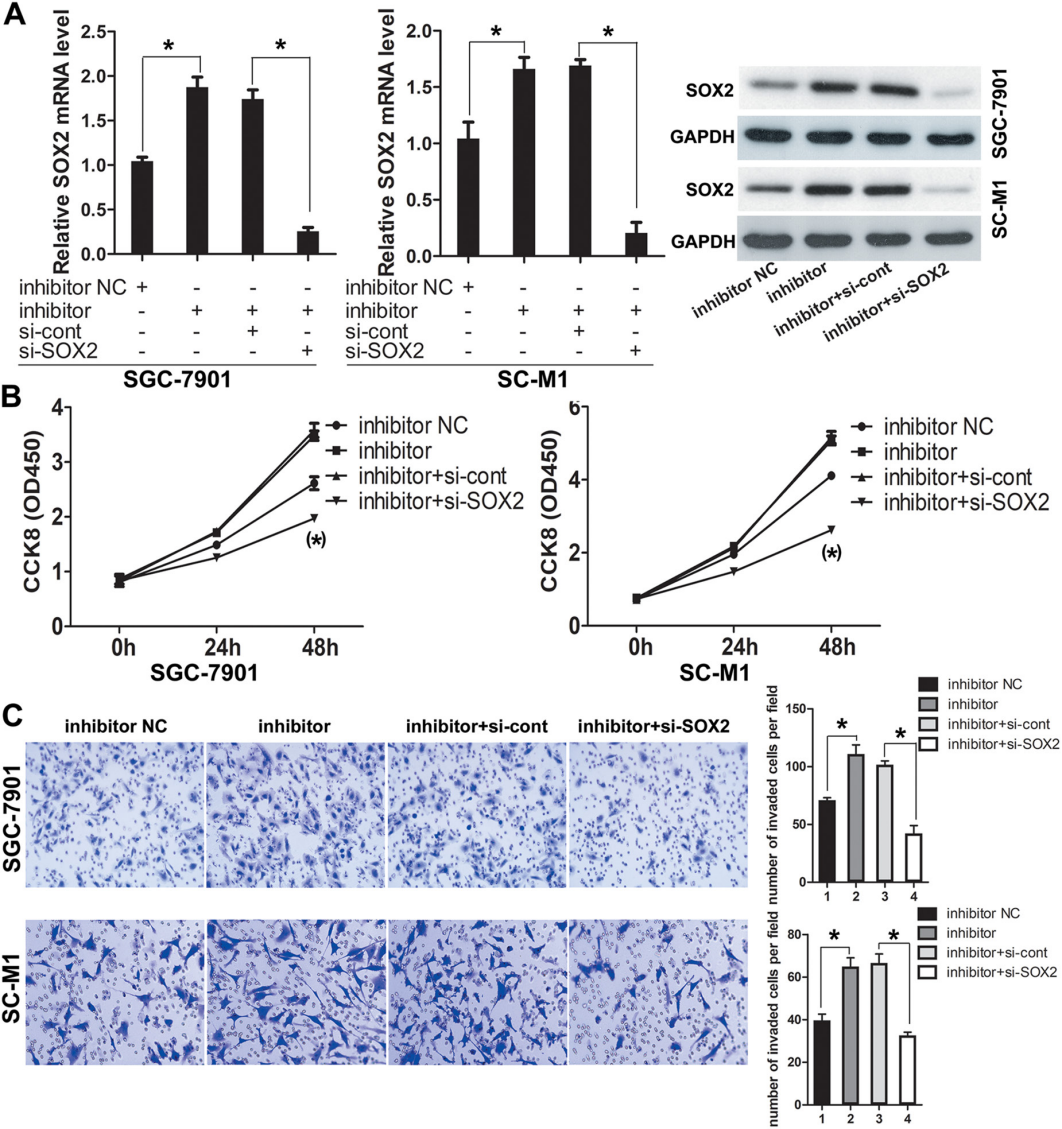
Knockdown of SOX2 antagonized the pro-invasion and pro-proliferation functions of miR-638 inhibitor **(A)** GC cells were co-transfected with miR-638 inhibitor, siRNA-SOX2 and the relative control. The transfection efficiency was confirmed by using qRT-PCR and Western blot. **(B)** After GC cells were co-transfected with miR-638 inhibitor and siRNA-SOX2, cell proliferation was measured using the CCK-8 assay at the indicated time points, and a growth curve was plotted. **(C)** Transwell assay was performed after GC cells were co-transfected with miR-638 inhibitor and siRNA-SOX2. The representative images of invasive cells at the bottom of the membrane stained with Giemsa were presented as shown. The quantifications of cell invasion were presented as numbers of invaded cells per field, magnification, ×20. (^*^ indicates p < 0.05).

Meanwhile, we took the cells transfected with miR-638 inhibitor and cells co-transfected with miR-638 inhibitor and siRNA-control as the control groups. The transfection efficiency was confirmed by qRT-PCR and Western blot. Results showed that the expression level of SOX2 was obviously decreased in cells (SC-M1 and SGC-7901) co-transfected with miR-638 inhibitor and siRNA-SOX2 compared to the control group (p<0.05, Figure 5A).

After that, we used the CCK-8 assay and Transwell invasion assay to confirm the role of SOX2 in GC cells by silencing SOX2. It revealed that silencing of SOX2 by siRNA could suppressed the promotion of cells proliferation and invasion induced by down-regulated miR-638. (p<0.05, Figure 5B and 5C).

## DISCUSSION

Existing studies about GC and miRNAs show that miRNAs play important roles in different progress of GC by multifarious mechanisms. For example, hypoxia-inducible microRNA-224 promotes the GC cell growth, migration and invasion by directly targeting RASSF8 [15], but miR-34a can act as a tumor suppressor targeting IGF2BP3 in gastric carcinogenesis [16]. Besides, miR-16 and miR-939 were confirmed to be contributed to the chemotherapy response of GC [17, 18].

As one of the microRNAs, miR-638 has been found to be a tumor suppressor in some cancers, such as breast cancer [19], acute myeloid leukemia (AML) [20], non-small-cell lung cancer (NSCLC) [21], and digestive system neoplasms including gastric caner [13, 14], liver cancer [22, 23] and colon cancer [24]. But Bhattacharya A et al. reported that miR-638 could protect melanoma cells from apoptosis and autophagy and promote melanoma metastasis [25]. Ren Y et al also found miR-638 acting as an oncogene in esophageal squamous cell carcinoma (ESCC) and breast cancer [26], paradoxical with Tan X et al. [19]. In GC, miR-638 has been reported down-regulated in cancer tissues and could suppress GC cell proliferation by targeting Sp2 and cyclinD1 [13, 14]. But the role of miR-638 in GC still needs to be confirmed, and more specific mechanism should be illuminated.

Herein, we confirmed that miR-638 expression is down-regulated in human GC tissues compared to adjacent tissues. This is consistent with the results of previous miRNA microarray and other studies [11–14]. And in GC cell lines (SGC-7901 and SC-M1), aberrant expression of miR-638 was related to the cell proliferation and invasion. We also found that SOX2 had a negative correlation with miR-638 in GC tissues, and miR-638 overexpression could decrease SOX2 expression level by directly binding the 3’-UTR of SOX2. *in vitro*, down-regulating SOX2 by siRNA could counteract the effect of miR-638 inhibitor on GC cells proliferation and invasion.

From the result of correlation analysis between miR-638 expression level and clinicopathological characteristics, there was no significantly correlation between tumor staging and miR-638 expression. In view of this result, enlarging sample volume and conducting experiments *in vivo* are beneficial strategies. More than that, other mechanisms besides cell cycle arrest contributing to tumor growth need to be further examined. In this study, miR-638 was not found to affect GC cells apoptosis (result not shown). However, in other studies, miR-638 was found to protect melanoma cells from apoptosis and autophagy [25], and promote starvation- or rapamycin-induced autophagy in ESCC and breast cells (KYSE450 and MCF-7) [26].

SOX2, a member of SRY-related HMG-box transcription factors families, is a well-established regulator of cell differentiation and development [27, 28]. More than that, accumulating studies suggest that SOX2 acts as an oncogene in some cancers, such as skin squamous-cell carcinoma [29, 30], lung squamous-cell carcinoma [31, 32], ovarian carcinoma [33], osteosarcomas [34] and glioblastoma [35].

Our study showed that SOX2 acted as an oncogene. Up-regulation of SOX2 caused by miR-638 inhibitor promoted GC cells proliferation and invasion *in vitro*, and silencing of SOX2 prevented GC cells proliferation and invasion *in vitro*. This result was in consistence with others. For instance, attenuated miR-638-SOX2 axis was reported to promote the invasion by inducing epithelial-mesenchymal transition in non-small-cell lung cancer [21], colorectal carcinoma [36], hepatocellular carcinoma [22].

Remarkably, Vanner and Boumahdi et al. revealed SOX2 was important in regulating cancer stem-like cells [30, 37]. SOX2-expressing cells could serve as the founding population responsible for tumor initiation, growth, and metastasis. So it is promising to illuminate the mechanism underlying miR-638 and SOX2 on the proliferation and invasion of GC cells. This provides us new ways for future therapeutic interventions in GC.

## MATERIALS AND METHODS

### Patients and specimens

From 2010 to 2014, patients with GC who underwent surgery for GC at the First Affiliated Hospital of Soochow University, Jiangsu Province, China, were recruited to this study. Fresh GC tissues and paired adjacent noncancerous tissues were collected from these patients. The diagnosis of GC was confirmed by histological examination by 2-3 pathologists who were blinded to the results, and the pathological stage of the cancer tissues was predicted according to the revised International System. Relevant clinical and pathological information was obtained from the hospital database. Patients who provided samples also provided written, informed consent to participate in this study. The Clinical Research Ethics Committee of Soochow University approved the study protocol and the consent procedure.

### Cell lines

Human GC cell line SGC-7901 and SC-M1 was purchased from the Institute of Biochemistry and Cell Biology of the Chinese Academy of Sciences (Shanghai, China). The cells were cultured in RPMI-1640 medium (Gibco, USA) with 10% fetal bovine serum (Gibco, USA) containing 100 U/mL penicillin and 100 mg/mL streptomycin (Gibco, USA) at 37°C in air containing 5% CO2.

### Transient transfection

The NC oligonucleotides (miR-control), miR-638 mimics (hsa-miR-638 mimics), NC inhibitor (anti-miR-control), and hsa-miR-638 inhibitor (anti-miR-638) were designed and synthesized by RiboBio (Guangzhou, China). The cells were seeded in six-well plates at 50% confluence one day before transfection. Cells were transfected with oligonucleotides using Invitrogen Lipofectamine®2000 (Thermo Fisher Scientific, Inc.) at a final concentration of 50 nM. The transfection efficiency was detected by qRT-PCR.

### Isolation of total RNA and quantitative RT-PCR

The total RNA extracted from the tumor tissues, paired adjacent noncancerous tissues, and cell lines using TRIzol (Sigma, USA). The RNA was then reverse transcribed to cDNA using RevertAid First Strand cDNA Synthesis Kit (Thermo Fisher Scientific, Inc). MiRNA expression levels were analyzed by TaqMan stem-loop qRT-PCR with a miRNA detection kit (Haoqin, ShangHai, China). Small nuclear RNA (snRNA) U6 was used to normalize data. The relative expression levels of SOX2 mRNA were examined by SYBR Green quantitative real-time PCR (TaKaRa, Japan) and normalized to the expression levels of β-actin. qRT-PCR was performed by using the ABI StepOnePlus Real-Time PCR system (ABI, CA, USA).

### Cell cycle profiling assay

Cells that were transfected with miRNA oligonucleotides were plated in the wells of six-well plates, incubated for 48 h, and then harvested for cell cycle distribution analysis. The cells were washed twice with phosphate-buffered saline, and fixed in 75% ethanol at 4°C overnight. The cells were washed again, incubated with RNase A, and stained with propidium iodide using Cell Cycle and Apoptosis Testing Kit (Beyotime, China) according to the manufacturer's protocol. The DNA content was detected with a Beckman Coulter flow cytometer, and the results were analyzed by ModFit LT for Windows Trial and Reader Version 4.0.

### CCK-8 assay

For the proliferation assay, GC cells that were transiently transfected for 24 h were replated in triplicate and tested for cell proliferation at different time points using the CCK-8 kit (Dojindo, Japan). Briefly, 3000 cells were seeded into each well of 96-well plates, incubated with 10 μL of CCK-8 for 3 h at 37°C, and the optical density was read at 450 nm with a microplate reader (Biotek).

### Invasion assay

BioCoat Matrigel (BD Biosciences, CA, USA) and invasion chambers (8-um size; Millipore, Germany) were used for the Transwell assay, according to the manufacturer's instructions, after cells were transfected with miRNA oligonucleotides for 24 h. Cells that had invaded the reverse face of the membrane were fixed in 4% paraformaldehyde and stained with Giemsa (Solarbio, China). A set of images was acquired using NIS Elements image analysis software (Nikon, Japan). The cells were counted under a light microscope in eight randomly selected fields.

### Luciferase reporter assay

The predicted miR-638 binding sequences in the 3’-UTR of SOX2 mRNA were cloned in a pMir luciferase reporter vector (Applied Biosystems, Carlsbad, CA, USA). The 3’-UTR fragment with mutant sequence in the predicted target site was cloned as a control group. Cells transfected miR-638 mimics or mimics-NC were plated into 24-well plate, and then wild type or mutant luciferase constructs were transfected into cells using Invitrogen Lipofectamine®2000 (Thermo Fisher Scientific, Inc.). For luciferase reporter assay, cells were collected 48 h after transfection and luciferase actions were measured using Luminoskan Ascent (Thermo Fisher Scientific, Inc.) following manufacturer's protocol.

### Western blot

Cells were washed in PBS and lysed in Cell lysis buffer P10013 for Western and IP (Beyotime, Nanjing, China), and total protein was quantified using Pierce BCA Protein Assay Kit (Thermo Fisher Scientific, Inc.). Then same amount of total cell lysates was resolved by SDS–polyacrylamide gel electrophoresis (PAGE), the protein were transferred to a polyvinylidene difluoride (PVDF) membrane (Millipore, Eschborn, Germany). The membrane were blocked in 5% NON-Fat Powdered Milk (BBI Life Sciences, Shanghai, China) for 1 h at room temperature and immunostained with SOX2 antibodies (1:500, santa, USA) at 4°C overnight. All results were visualized using Medical X-ray Processor (Kodak, New York, USA) and then analyzed by Quantity One Soft.

### Statistical analysis

Each experiment was repeated at least three times. The results are presented as the mean values ± standard error of the mean (SEM), and the data were analyzed using unpaired, two-tailed Student's t test or the χ^2^ test. P values <0.05 were considered statistically significant. Statistical analyses were performed by GraphPad Prism 6.0 software (GraphPad Software Inc., CA, USA). The accession numbers of has-miR-638 and SOX2 were MIMAT0003308 and NM_003106.2, respectively.
